# Trading certainty for speed - how much uncertainty are decisionmakers and guideline developers willing to accept when using rapid reviews: an international survey

**DOI:** 10.1186/s12874-017-0406-5

**Published:** 2017-08-14

**Authors:** Gernot Wagner, Barbara Nussbaumer-Streit, Judith Greimel, Agustín Ciapponi, Gerald Gartlehner

**Affiliations:** 1 Department for Evidence-based Medicine and Clinical Epidemiology, Danube University Krems, Cochrane Austria, Dr. Karl Dorrek Strasse 30, 3500 Krems, Austria; 2 University Hohenheim, Schloss Hohenheim 1, 70599 Stuttgart, Germany; 3 Institute for Clinical Effectiveness and Health Policy, Dr. Emilio Ravignani 2024, C1414CPV Buenos Aires, Argentina; 4 RTI International, 3040 Cornwallis Road, PO Box 12194, Research Triangle Park, NC 27709-2194 USA

**Keywords:** Systematic review, Rapid review, Guideline developer, Decisionmaker, Decision-making, Uncertainty

## Abstract

**Background:**

Decisionmakers and guideline developers demand rapid syntheses of the evidence when time sensitive evidence-informed decisions are required. A potential trade-off of such rapid reviews is that their results can have less reliability than results of systematic reviews that can lead to an increased risk of making incorrect decisions or recommendations. We sought to determine how much incremental uncertainty about the correctness of an answer guideline developers and health policy decisionmakers are willing to accept in exchange for a rapid evidence-synthesis.

**Methods:**

Employing a purposive sample, we conducted an international web-based, anonymous survey of decisionmakers and guideline developers. Based on a clinical treatment, a public health, and a clinical prevention scenario, participants indicated the maximum risk of getting an incorrect answer from a rapid review that they would be willing to accept. We carefully reviewed data and performed descriptive statistical analyses.

**Results:**

In total, 325 (58.5%) of 556 participants completed our survey and were eligible for analysis. The median acceptable incremental risk for getting an incorrect answer from a rapid review across all three scenarios was 10.0% (interquartile range [IQR] 5.0–15.0). Acceptable risks were similar for the clinical treatment (*n* = 313, median 10.0% [IQR 5.0–15.0]) and the public health scenarios (*n* = 320, median 10.0% [IQR 5.0–15.0]) and lower for the clinical prevention scenario (*n* = 312, median 6.5% [IQR 5.0–10.5]).

**Conclusions:**

Findings suggest that decisionmakers are willing to accept some trade-off in validity in exchange for a rapid review. Nevertheless, they expect the validity of rapid reviews to come close to that of systematic reviews.

**Electronic supplementary material:**

The online version of this article (doi:10.1186/s12874-017-0406-5) contains supplementary material, which is available to authorized users.

## Background

Systematic reviews have become an important source of information for decisionmakers because they provide scientifically rigorous, independent, and accurate syntheses of scientific evidence [1]. Well conducted systematic reviews employ high methodological standards to address clinical or healthcare policy questions by identifying, appraising, and summarizing primary research [2]. A drawback of systematic reviews, however, is that the amount of time they require to complete (a minimum of 6 months to 1 year) can be too long for the time-sensitive needs of decisionmakers [3]. As a consequence, rapid reviews have become a pragmatic alternative to systematic reviews [4].

The term *rapid review* refers to diverse approaches of evidence syntheses that produce results within a few weeks to a few months by simplifying some methodological steps or by omitting steps all together [4–8]. Although the definition of rapid reviews can vary, typically they are characterized by a strong focus on the specific needs of decisionmakers [9–12] and by methodological shortcuts. These shortcuts modify the most time-consuming steps of systematic reviews, in particular literature search and identification, quality assessment, and evidence synthesis [13]. For example, rapid reviews often limit searches to commonly used databases employing very specific search filters, omit dual data abstraction and critical appraisal and rarely conduct quantitative analyses [5].

To date, little research on the validity of rapid reviews has been conducted. Studies comparing the conclusions between rapid reviews and systematic reviews, in general, found them to be similar [5]. Nevertheless, because of methodological shortcuts, results of rapid reviews have the potential to be less reliable than those of systematic reviews [14]. Or in other words, uncertainty about the correctness of results could play a larger role in rapid reviews than in systematic reviews. This, in turn, could increase the risk of making incorrect decisions or recommendations.

Although uncertainty is an important and omnipresent issue in healthcare, no universally agreed upon definition of uncertainty exists. Concepts of uncertainty, however, always imply a subjective awareness about one’s lack of knowledge [15]. Han and colleagues distinguish among three sources of uncertainty in healthcare, namely probability (the likelihood of a future event), ambiguity (possible different states for a single event), and complexity (the multiplicity of causal factors and interpretive cues that make a phenomenon difficult to comprehend) [15]. The increased uncertainty in rapid reviews which results from streamlining methods might affect estimates of probabilities of individual outcomes and cause ambiguities. Rapid reviews do not affect, however, uncertainty due to the complexity of a medical question or healthcare situation.

When deciding whether or not to favor a rapid review as an alternative to a systematic review, decisionmakers have to weigh the celerity to complete a decision-support review against the potential risk of incorrect answers for some outcomes of interest.

The aim of our study was to determine how much incremental risk of getting an incorrect answer that guideline developers and health policy decisionmakers are willing to accept in exchange for an evidence-synthesis that can be obtained and utilized more expediently than a full systematic review.

## Methods

We conducted an international web-based survey in English, German, and Spanish targeting decisionmakers and guideline developers who might commission evidence syntheses to inform their decisions and recommendations. Throughout the manuscript we adhere to the Checklist for Reporting Results of Internet E-Surveys (CHERRIES) (Additional file 1) [16].

### Development of the survey

The development of the survey was guided by principles for web surveys as proposed by Dillman et al. [17]. To determine the level of incremental risk of getting an incorrect answer that participants are willing to accept, we created three different hypothetical scenarios (presented in Table 1). A systematic review which could provide 100% certainty (a hypothetical assumption) would take 18 months to complete. A rapid review on the same topic could be finished within 3 months but would carry a certain risk of providing an incorrect answer regarding efficacy, effectiveness, and safety, which could lead to a wrong decision or recommendation. We purposely picked these time frames for rapid and systematic reviews so that “waiting” for the systematic review would not be a viable option. A 100% certainty is hypothetical and will be lower based on the available evidence of a certain outcome.

**Table 1:** Hypothetical decision-making scenarios used in the survey

Scenario	Medical field	Description
Scenario 1	Clinical Treatment	A new drug has the potential to heal a chronic infectious disease (prevalence 3%) for which no cure has been available to date. The drug is extremely expensive (US$ 84,000 per course of treatment, approximately US$ 50,000 per quality-adjusted life year gained), and it does not work for all genotypes of the infectious agent. Furthermore, it can lead to serious side effects in rare cases.
Scenario 2	Public Health Intervention	A new vaccination has the potential to prevent a particular type of cancer (incidence 9.9/100,000 per year), but no long-term studies showing the effectiveness are available to date. Preliminary data on the reduction of infection rates of the cancer-causing virus are promising. Interest groups are pushing heavily for health officials to recommend the vaccine and for insurance plans to cover the costs. The costs of a population-wide vaccination campaign would be substantial (US$ 43,600 per quality-adjusted life year gained).
Scenario 3	Clinical Prevention	A drug class has been widely prescribed for the primary and secondary prevention of cardiovascular disease. The number needed to treat to prevent one cardiovascular event is 71 (over 10 years at a cost of € 35,000 per quality-adjusted life year gained). Several new drugs within this class have been approved recently. They are heavily marketed by the industry but, despite higher costs, whether they have any therapeutic benefit compared with that from older drugs remains unclear.

€ = Euro**;** US$ = United States Dollar

The survey asked participants to quantify the maximum risk of getting an incorrect answer that they would be willing to accept in exchange for a rapid synthesis for each of the three scenarios. For example, choosing 20% as the maximum risk of getting an incorrect answer would mean that rapid reviews are an acceptable form of decision support for the participant if results coincide with a systematic review 80% of the time. We asked participants to indicate the maximum acceptable risk on a visual scale ranging from 0 to 50%. A 50% risk would mean that flipping a coin would produce the same proportion of correct and incorrect answers. Participants had the option not to answer a scenario if they felt that the topic was beyond their area of expertise.

In addition, participants provided personal characteristics including country of residence, gender, and age. They also specified how the evidence is utilized in their decision-making processes. The entire survey and the recruitment e-mail in English are available in Additional files 2 and 3.

We pilot-tested the survey with guideline developers, methodologists, and researchers experienced in using evidence syntheses. Those data were not used for final analyses. We programmed the survey to exclude anyone who indicated at the start that they do not use evidence syntheses for decision-making and guideline development. For those respondents the survey automatically ended.

### Study sample and data collection

We conducted the survey from April to July 2016 using LimeSurvey 2.0 (www.limesurvey.org) as an electronic web-based survey tool. We used a nonrandom purposive sample [18] of guideline developers and healthcare decisionmakers, mostly from regulatory agencies, health insurance companies, or health policy groups. We identified possible participants through professional networks and associations. Overall, we contacted 57 organizations (e.g., Cochrane Centers, Guidelines International Network [GIN], US Consumers Union, World Health Organization [WHO], American College of Physicians [ACP], Center for Disease Control and Prevention [CDC]). Contact occurred directly or through referral from other respondents (snowballing) via e-mail with a link to the survey. Participants were asked to complete the survey and forward the survey-link to eligible colleagues. We also contacted representatives of relevant organizations and networks to distribute the survey within their organizations and their countries, respectively. We did not offer any incentives for participation. Reminder notifications were sent by e-mail 2 and 4 weeks after the initial contact. Once a participant completed the survey, an electronic cookie prevented multiple submissions from the same computer. All responses were anonymous. Data were stored securely and were protected from unauthorized access. Participants who were interested in survey results were able to submit their e-mail addresses. The Danube University Institutional Review Board determined that ethics approval was not necessary for an anonymous web survey.

### Statistical analysis

At the conclusion of the survey, we carefully reviewed and cleaned all data. We performed descriptive statistical analyses and present categorical data as absolute frequencies and percentages; continuous data as the median, the 5th, 25th, 75th, and 95th percentiles, and the range. We conducted all statistical analyses using STATA 14.2 (StataCorp, College Station, TX, USA).

## Results

Overall, 350 (63%) out of 556 participants completed the survey. We excluded responses from 16 participants who indicated that they do not use evidence-syntheses for decision-making or guideline development and were redirected to the end of the survey even before answering the scenarios. In addition, we excluded 9 participants who opted-out of all three scenarios, leaving 325 (59%) remaining eligible participant responses. Most of the participants (*n* = 136, 42%) used the English version of the survey. Detailed demographic characteristics of survey participants are presented in Table 2. Nearly half of the participants specified a European country of residence. The majority of participants were decisionmakers (Table 3).

**Table 2:** Characteristics of participants of the survey

Participant characteristics (number of responses)	Number of participants (% of response)
Number of participants	
Total	556 (100)
Completed survey	350 (62.9)
Ineligible (Do not use evidence syntheses for decision-making purposes)^a^	16
Ineligible (Opt-out for all three scenarios)	9
Eligible	325 (58.5)
Gender (*n* = 323)^b^	
Female	165 (51.1)
Male	158 (48.9)
Age – years (*n* = 323)^b^	
21 – 30	15 (4.6)
31 – 40	75 (23.2)
41 – 50	92 (28.5)
51 – 60	109 (33.8)
61 – 70	29 (9.0)
> 70	3 (0.9)
Selected survey language (*n* = 325)	
English	136 (41.9)
Spanish	104 (32.0)
German	85 (26.1)
Type of user of evidence (*n* = 325)^c^	
Guideline developer	143 (44.0)
Health policy decisionmaker	68 (20.9)
Decisionmaker in a health insurance company	41 (12.6)
Decisionmaker in a regulatory agency	19 (5.9)
Hospital administrator	16 (4.9)
Other	120 (36.9)
Residence by continents with most commonly reported countries (*n* = 325)	
Europe	147 (45.2)
Austria	53 (16.3)
United Kingdom	47 (14.5)
Germany	31 (9.5)
South and Central America	114 (35.1)
Argentina	45 (13.9)
Peru	18 (5.5)
Colombia	17 (5.2)
North America	57 (17.5)
Canada	33 (10.2)
United States of America	24 (7.4)
Africa	3 (0.9)
Australia and New Zealand	3 (0.9)
Asia	1 (0.3)

*n* number of participants

^a^Participants were redirected to the end of the survey even before answering the scenarios

^b^Not reported by two participants; ^c^Participants could select more than one option

**Table 3:** Risk of getting an incorrect answer that participants are willing to accept according to type of evidence user and scenario

Types of user of evidence	Acceptable risk (%)							
	N^a^	Median	p25	p75	p5	p95	Min	Max
All participants (*n* = 325)								
All scenarios	945	10	5	15	1	30	0	50
Clinical Treatment (Scenario 1)	313	10	5	15	1	30	0	50
Public Health Intervention (Scenario 2)	320	10	5	15	1	30	0	50
Clinical Prevention (Scenario 3)	312	6.5	5	10.5	1	30	0	50
Guideline developers (*n* = 94)								
All scenarios	275	6	5	10	1	25	0	50
Clinical Treatment (Scenario 1)	91	5	5	10	1	20	1	38
Public Health Intervention (Scenario 2)	94	10	5	15	1	25	0	50
Clinical Prevention (Scenario 3)	90	5	5	10	1	25	1	42
Decisionmakers^b^ (*n* = 182)								
All scenarios	527	10	5	15	1	34	0	50
Clinical Treatment (Scenario 1)	175	10	5	15	1	30	0	50
Public Health Intervention (Scenario 2)	177	10	5	18	1	40	0	50
Clinical Prevention (Scenario 3)	175	10	5	15	0	40	0	50
Guideline developers and decisionmakers^b^ (*n* = 49)								
All scenarios	143	6	5	10	1	25	0	50
Clinical Treatment (Scenario 1)	47	8	5	15	1	30	0	50
Public Health Intervention (Scenario 2)	49	5	5	10	1	25	0	41
Clinical Prevention (Scenario 3)	47	5	2	10	0	25	0	50

*IQR* interquartile range, *Min* Minimum, *Max* Maximum, *N* number of participants

p5 = 5th percentile, p25 = 25th percentile, p75 = 75th percentile, p95 = 95th percentile

^a^Number of responses; participants had the option to not answer individual scenarios

^b^Including health policy decisionmaker, decisionmaker regulatory agency, decisionmaker health insurance company, hospital administrator and other types of evidence users. Participants could select more than one option

As outlined in the methods, participants had the option to not provide estimates for scenarios that they felt unfamiliar with. Twenty-one respondents opted-out of the clinical treatment scenario (scenario 1, Table 1), 14 of the public health scenario (scenario 2, Table 1), and 22 of the clinical prevention scenario (scenario 3, Table 1).

Across all three scenarios, we analyzed 945 responses. The median acceptable incremental risk of getting an incorrect answer from a rapid review was 10% (interquartile range [IQR] 5.0–15.0). Individual answers for the three scenarios, however, varied widely and ranged from 0% (rapid reviews have to be as reliable as systematic reviews) to 50% (rapid reviews are still useful, even if they provide incorrect answers in 5 out of 10 reviews). Table 3 provides descriptive statistics of answers overall, stratified by scenarios and user-types of evidence syntheses.

Regarding individual scenarios (Table 1), respondents’ willingness to accept risks for incorrect results was the same for the clinical treatment (scenario 1, *n* = 313) and the public health (scenario 2, *n* = 320) scenarios with a median acceptable risk of 10% (IQR 5.0% to 15% for both). For the clinical prevention scenario (scenario 3, *n* = 312), the median acceptable risk was 6.5% (IQR 5.0% to 10.5%). Across all three scenarios, male participants (*n* = 158) were more cautious in their willingness to accept incorrect answers than female (*n* = 165) participants (median 5% [IQR 5.0–12.0] vs. 10% [IQR 5.0–15.0]).

**Fig. 1:**
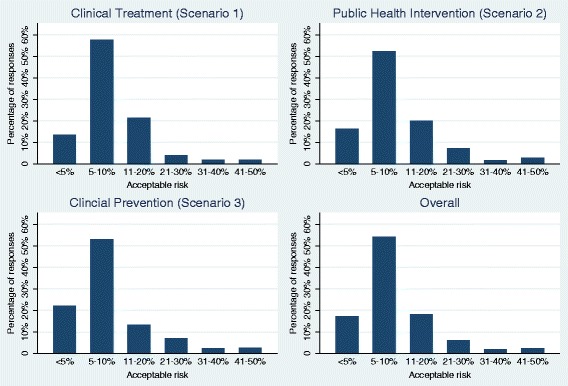
Acceptable risk for an incorrect answer overall and by scenario

**Fig. 2:**
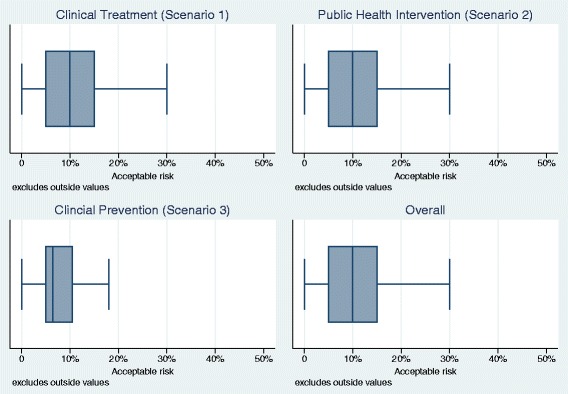
Box plots of acceptable risk for an incorrect answer overall and by scenario

Figure 1 presents bar charts of the responses for each scenario and across all three scenarios. Figure 2 depicts corresponding box plots. The box represents 50% of the answers of the survey (i.e., the IQR); horizontal lines at both ends of the boxes represent the remaining 50% of data without extreme outliers. The vertical line in the box represents the median. The left end of the box indicates first quartile, the right end the third quartile of respondent answers regarding the maximum acceptable risk.

The overall acceptable risk for getting an incorrect answer was, in general, similar across types of evidence users (Table 3).

## Discussion

To the best of our knowledge, this is the first study exploring the risk of getting an incorrect answer that healthcare decisionmakers and guideline developers are willing to accept as a trade-off for using rapid reviews. Participants of our survey, on average, viewed 10% as the maximum tolerable risk of getting an incorrect answer from a rapid review. In other words, respondents of our survey expect rapid reviews to provide answers similar to systematic reviews in at least nine out of ten cases.

Because rapid reviews employ heterogeneous methodological approaches [3, 5, 8–10], assessing the validity is difficult. Recent research has focused on the impact of distinct changes to systematic review methods such as abbreviated literature searches and their impact on results. For example, several methodological studies have shown that abbreviated search strategies do not detect the same number of studies that comprehensive systematic searches do [19–23]. The impact of studies that were missed on effect estimates of meta-analyses, however, was small [24]. An ongoing methods project uses a non-inferiority and meta-epidemiological approach to assess whether the reduced sensitivity of abbreviated searches has an impact on conclusions of Cochrane reviews, not only on effect estimates [25].

Because every decision-making process is more complex than relying exclusively on a synthesis of the evidence, we had to make several assumptions to simplify the scenarios that we presented to participants. First, we made the hypothetical assumption that systematic reviews can provide 100% certainty for decision making. In reality, systematic reviews are an imperfect gold standard that is susceptible to random and systematic errors just as any other study design. Because we were interested in the incremental risk of getting an incorrect answer from rapid reviews, we had to employ a hypothetical perfect reference standard. Furthermore, we deliberately disregarded that any decision-making is context-specific and that evidence usually is only one of several factors that contribute to a decision process. These are artificial scenarios and timeframes that simplify the contexts of real-world decisions.

In addition to these assumptions, our study has several other limitations. First, we used a purposive sample of health policy decisionmakers and guideline developers who use evidence-synthesis for decision-making. Random sampling was not possible because we were unable to define the entire global population of decisionmakers. Although we reached participants from 33 countries, as any nonrandom sample, our results might be influenced by exclusion bias which potentially limits the generalizability. Whether or not our findings have been swayed by nonresponse due to survey fatigue, remains unclear. We cannot determine the proportions of nonresponse because we do not know the exact number of people who received the request to participate with the snowballing technique.

Second, it remains unclear how generalizable our findings are to health decisions in general because only participants who demand rapid reviews for decision-making or guideline development were asked to indicate the acceptable degree of uncertainty based on three distinct, hypothetical scenarios. We were not able to cover all fields and scenarios of clinical care and health policy where timely decisions are required. In particular, decisionmakers in certain medical fields may not accept any risk of getting an incorrect answer from rapid reviews. Furthermore, we did not evaluate perspectives of clinicians and patients with respect to evidence-informed decisions, because we were interested primarily in opinions of decisionmakers who might commission systematic or rapid reviews. Based on the characteristics of participants, we are confident that our sample reflects this target population.

Finally, we cannot rule out a certain extent of measurement error because respondents may have misunderstood our questions. Some dispersion of answers could be attributable simply to a misunderstanding or misinterpretation of the questions. For example, the term ‘certainty’ is used by the GRADE (Grading of Recommendations Assessment, Development and Evaluation) Working Group to describe the level of certainty of results from a synthesis of evidence which is conceptually different from our use of the term ‘certainty’. Nevertheless, when we discarded responses that were outliers in sensitivity analyses, the overall findings did not change.

Rapid review methodological research is a dynamic field and a topic of great international interest. Different types of evidence users demand rapid reviews for decision-making and recommendations. For end-users trade-offs are of particular interest. Further research is needed to determine the reliability of rapid reviews by evaluating if different streamlined methods are non-inferior to a comprehensive, systematic literature review approach.

Rapid reviews as a method of evidence synthesis are unlikely to be more valid than systematic reviews. The crucial question is which methodological approaches yield the smallest trade-offs in validity to determine which approaches are not substantially worse than systematic reviews. Such an assessment of non-inferiority, however, requires a judgement regarding a margin in which the observed difference in validity is irrelevant. Our findings that decisionmakers are willing to accept a maximum incremental risk of getting a wrong answer of 10% provides an important first step that can inform research about the comparative validity of rapid reviews and systematic reviews. To date, however, the available methods research is still insufficient to draw conclusions on whether or not rapid reviews can achieve such a threshold. Future methods studies need to explore which methodological shortcuts provide the best trade-off between certainty and speed.

The recently established Cochrane Rapid Review Methods Group will play an important role regarding guidance for production of rapid review products, conduct methodological research and provide a platform for discussion and training [26].

## Conclusion

Findings suggest that decisionmakers and guideline developers are willing to accept some trade-off in validity in exchange for a rapid synthesis of the evidence. Nevertheless, they expect the validity of rapid reviews to come close to that of systematic reviews. Future research needs to determine the impact of specific methodological shortcuts on the validity of rapid reviews and its consequences on conclusions of decisionmakers.

## Additional files

Additional file 1: CHERRIES Checklist. This file contains the Checklist for Reporting Results of Internet E-Surveys (CHERRIES) with a description of all items addressed in this survey. (DOCX 22 kb)

Additional file 2: Recruitment e-mail. This file contains the text of the recruitment e-mail in English for individually contacted decisionmaker and guideline developer. (DOCX 16 kb)

Additional file 3: Questionnaire in English. This file contains screenshots of the questionnaire in English. (DOCX 709 kb)

## Abbreviations

€: Euro
ACP: American College of Physicians
CDC: Center for Disease Control and Prevention
GIN: Guidelines International Network
IQR: interquartile range
Max: Maximum
Min: Minimum
n: number of participants
p25: 25th percentile
p5: 5th percentile
p75: 75th percentile
p95: 95th percentile
US$: United States Dollar
WHO: World Health Organization
