# Supplementary material for: Trading certainty for speed - how much uncertainty are decisionmakers and guideline developers willing to accept when using rapid reviews: an international survey
Source: BMC Med Res Methodol. 2017 Aug 14;17:121. doi: 10.1186/s12874-017-0406-5 (PMC5557322; doi:10.1186/s12874-017-0406-5)
Supplement: Supplementary file 1 — CHERRIES Checklist. This file contains the Checklist for Reporting Results of Internet E-Surveys (CHERRIES) with a description of all items addressed in this survey. (DOCX 22 kb) [file 12874_2017_406_MOESM1_ESM.docx]

**Additional file 1**

**CHERRIES checklist for the international survey “How much uncertainty are decisionmakers and guideline developers willing to accept when using rapid reviews?”**

| ***Item Category*** | ***Checklist Item*** | ***Description*** |
| --- | --- | --- |
| **Design** | Survey design | We conducted an international web-based survey in English, German, and Spanish targeting decisionmakers and guideline developers who might commission evidence summaries to inform their decisions and recommendations. We used a nonrandom purposive sample of guideline developers and healthcare decisionmakers. |
| **IRB (Institutional Review Board) approval and informed consent process** | IRB approval | The Danube University Institutional Review Board determined that ethics approval was not necessary for an anonymous web survey. |
|  | Informed consent | Not applicable |
|  | Data protection | Data were stored securely and were protected from unauthorized access. |
| **Development and pre-testing** | Development and testing | We pilot-tested the survey with guideline developers, methodologists and researchers experienced in using evidence syntheses. |
| **Recruitment process and description of the sample having access to the questionnaire** | Open versus closed survey | Open survey |
|  | Contact mode | Contact occurred directly or through referral from other respondents (snowballing) via e-mail with a link to the survey. Participants were asked to complete the survey and forward the survey-link to eligible colleagues. We also contacted representatives of relevant organizations and networks to distribute the survey within their organizations and their countries, respectively. |
|  | Advertising the survey | The survey was announced in newsletters and mailings of relevant organisations |
| **Survey administration** | Web/E-mail | E-mail with a link |
|  | Context | Electronic web-based survey tool (LimeSurvey 2.0, [www.limesurvey.org](http://www.limesurvey.org)) |
|  | Mandatory/voluntary | Voluntary survey |
|  | Incentives | We did not offer any incentives for participation. |
|  | Time/Date | Data were collected from April to July 2016 |
|  | Randomization of items or questionnaire | Not performed |
|  | Adaptive questioning | We programmed the survey to exclude anyone who indicated at the start that they do not use evidence summaries for decision-making and guideline development. For these respondents the survey automatically ended. |
|  | Number of Items | Page 1: introduction, Page 2: 1 item  Page 3-5: 2 items each, Page 6: 3 items  Page 7: 1 item |
|  | Number of screens (pages) | 7 screens |
|  | Completeness check | We checked data for completeness. |
|  | Review step | Respondents had the possibility to review and change their answers through a back button. |
| **Response rates** | Unique site visitor | Not available |
|  | View rate (Ratio of unique survey visitors/unique site visitors) | Not available |
|  | Participation rate (Ratio of unique visitors who agreed to participate/unique first survey page visitors) | Not available |
|  | Completion rate (Ratio of users who finished the survey/users who agreed to participate) | 350 out of 556 completed (62.9%) |
| **Preventing multiple entries from the same individual** | Cookies used | Yes |
|  | IP check | No |
|  | Log file analysis | No |
|  | Registration | Not applicable |
| **Analysis** | Handling of incomplete questionnaires | Incomplete questionnaires were not eligible for analysis. |
|  | Questionnaires submitted with an atypical timestamp | Not applicable |
|  | Statistical correction | No |
