# Supplementary figures and images for: Trading certainty for speed - how much uncertainty are decisionmakers and guideline developers willing to accept when using rapid reviews: an international survey
Source: BMC Med Res Methodol. 2017 Aug 14;17:121. doi: 10.1186/s12874-017-0406-5 (PMC5557322; doi:10.1186/s12874-017-0406-5)

**Additional file 3**

**Questionnaire in English**


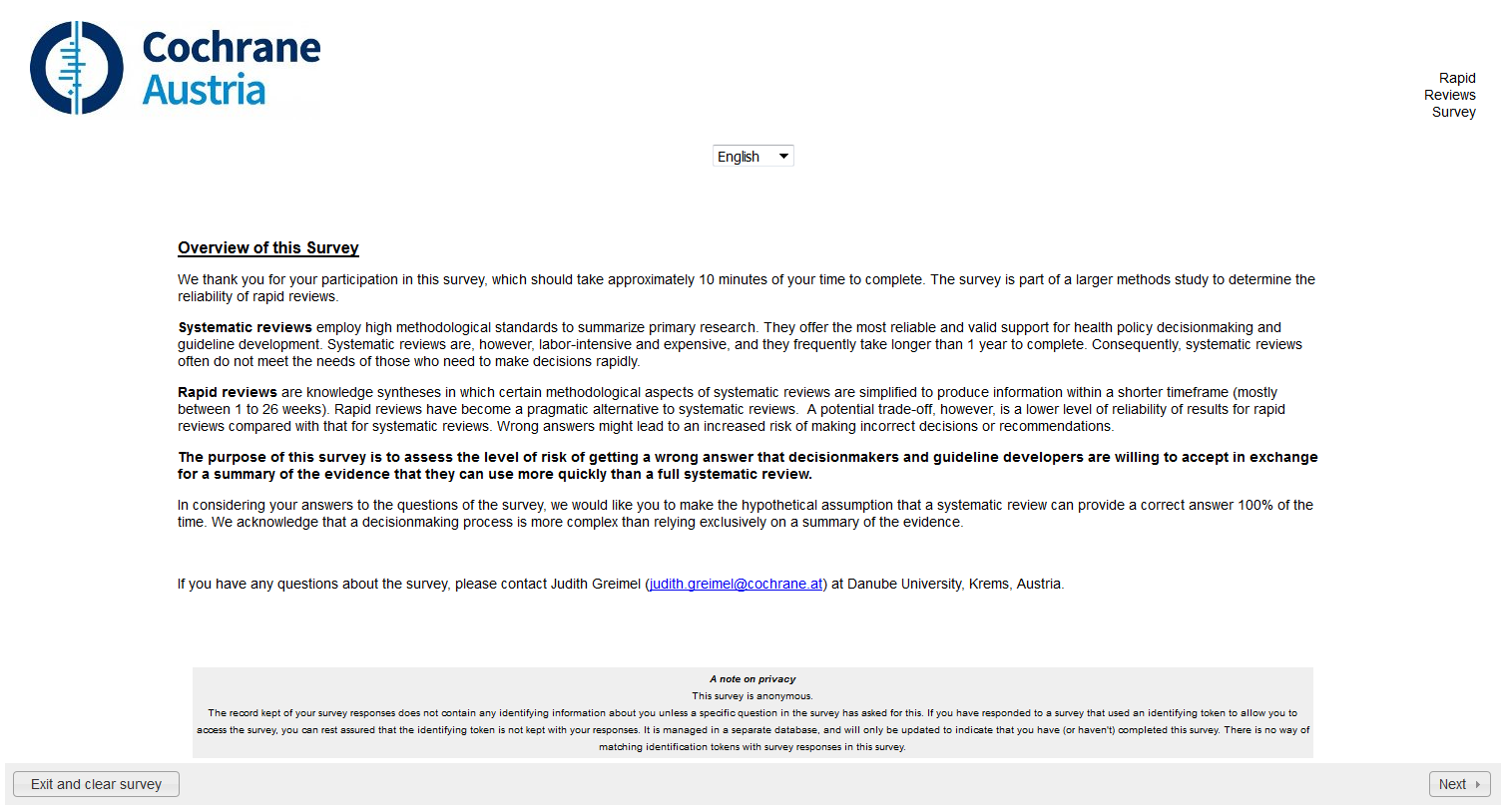


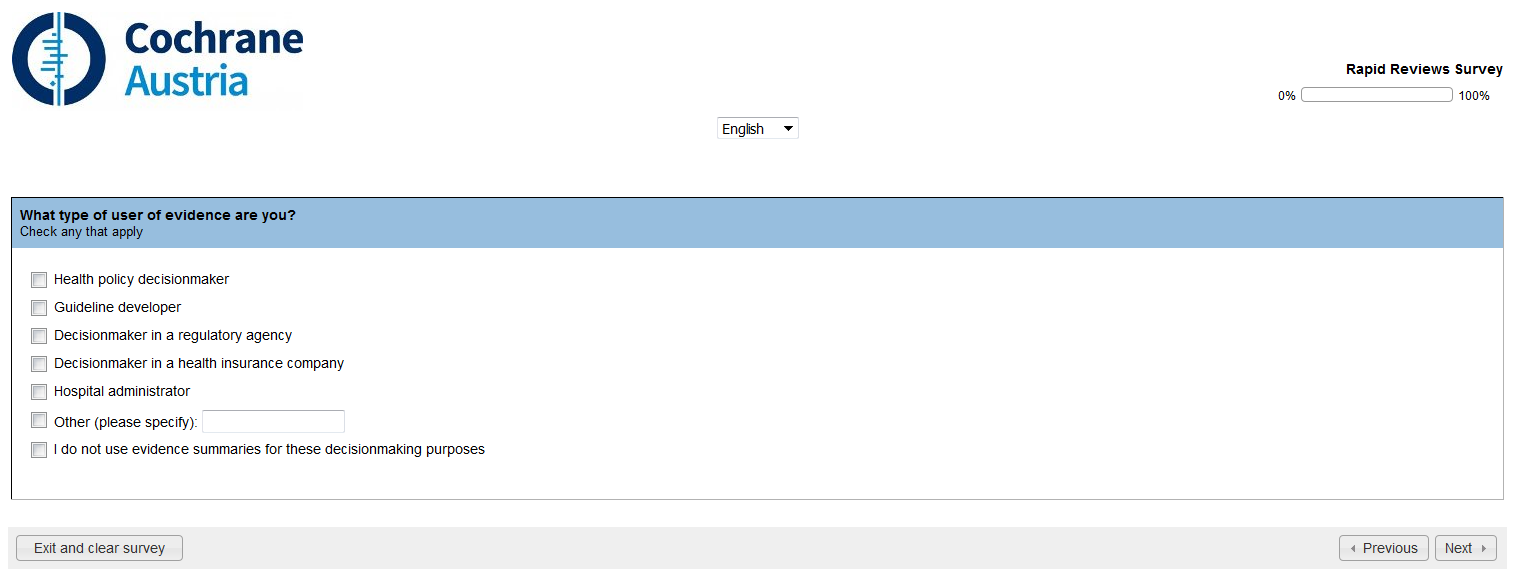


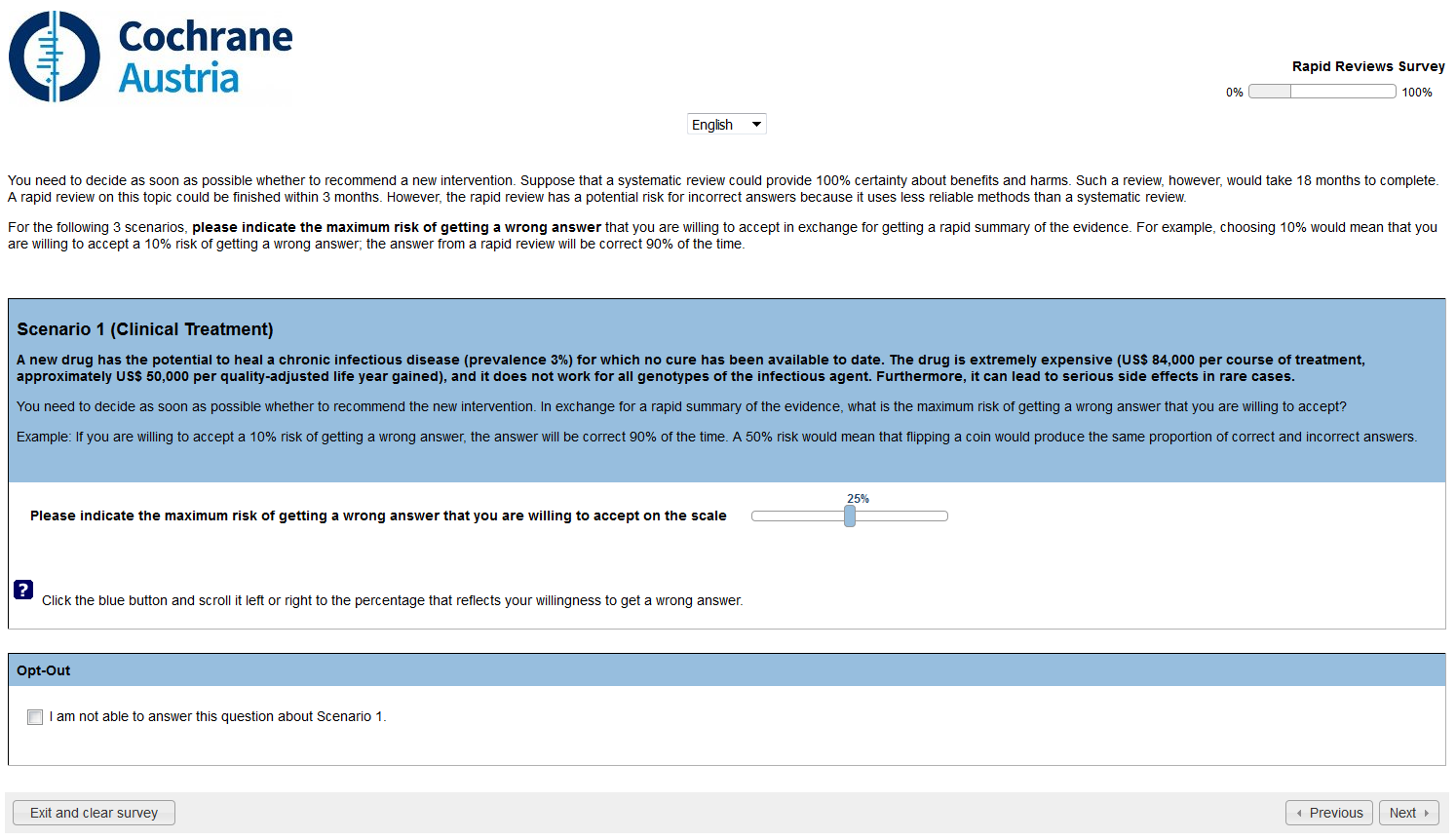


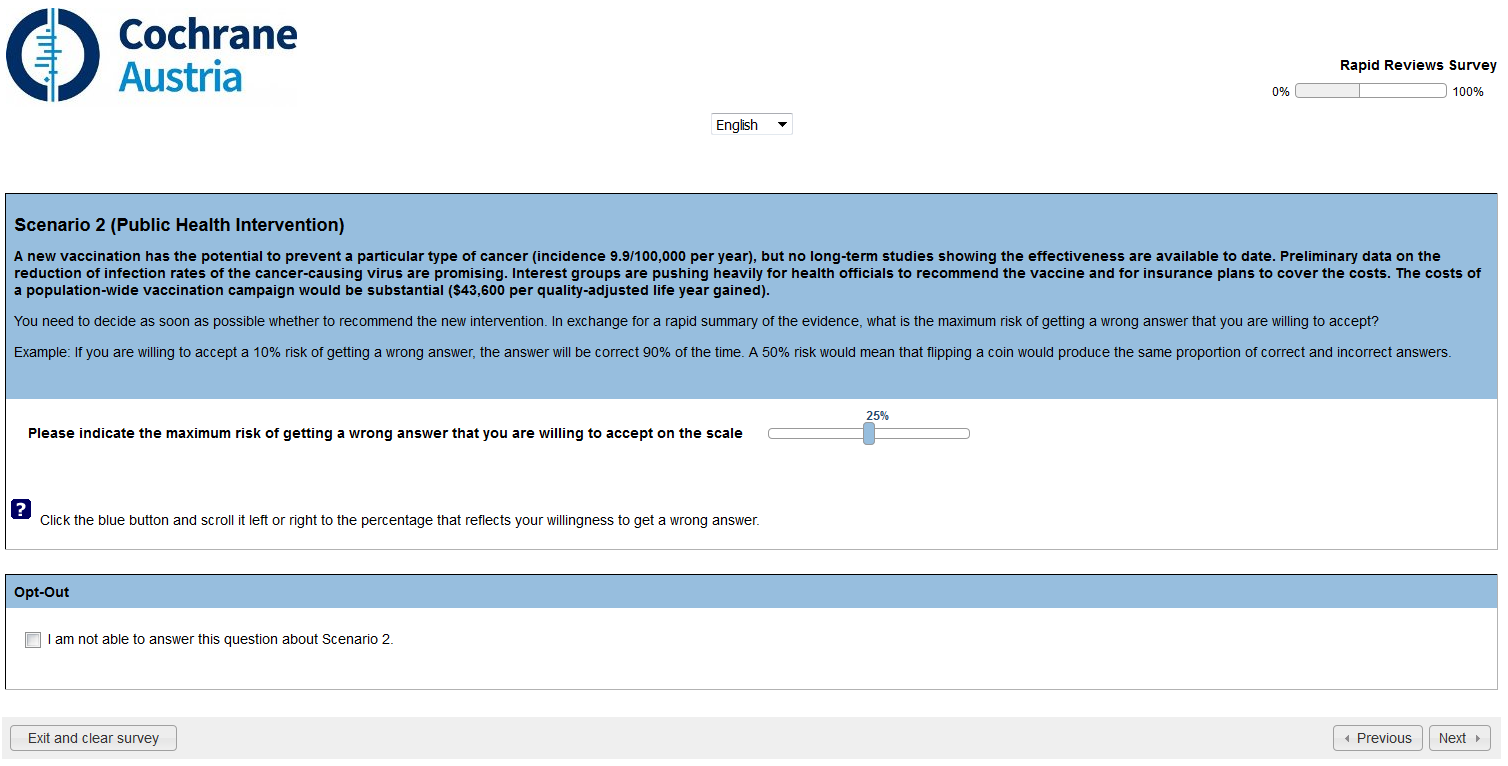


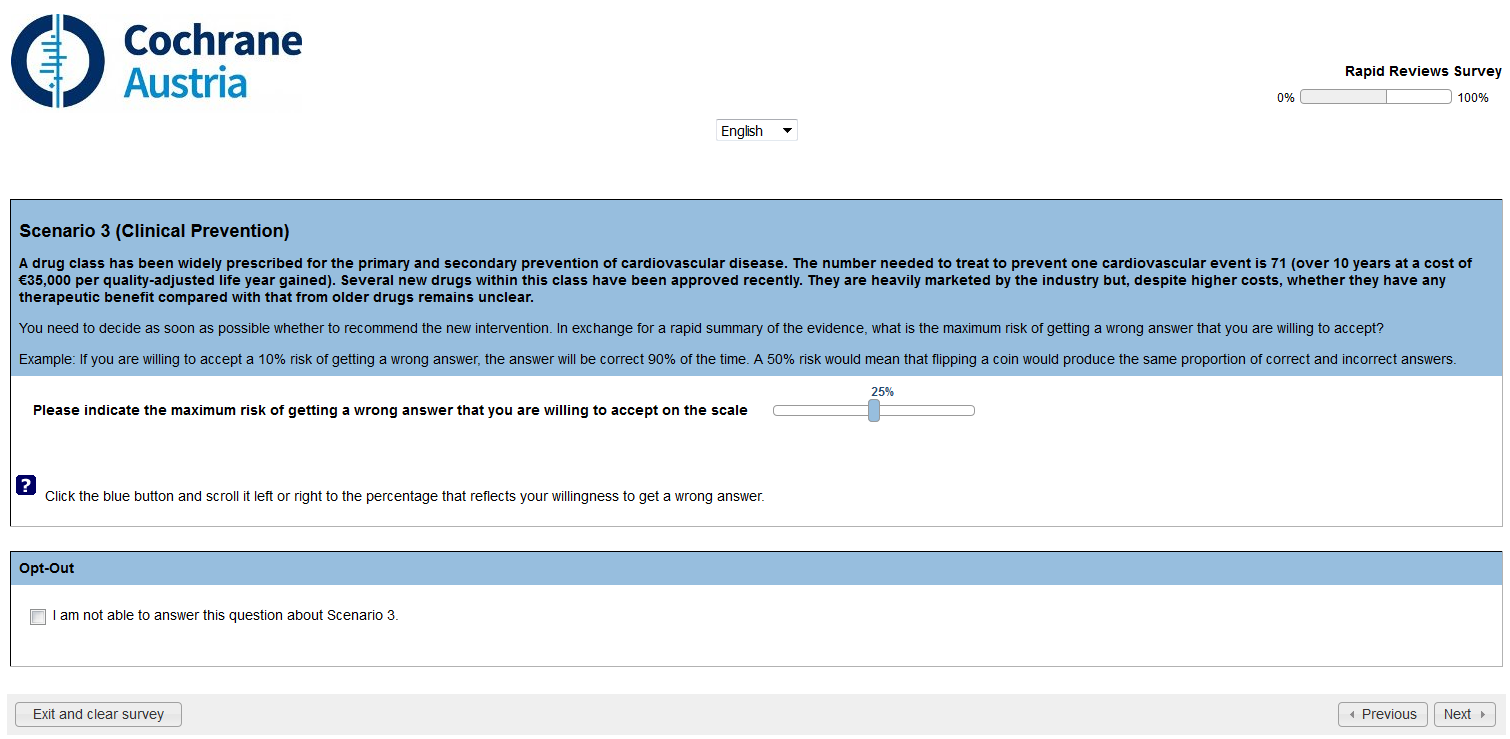


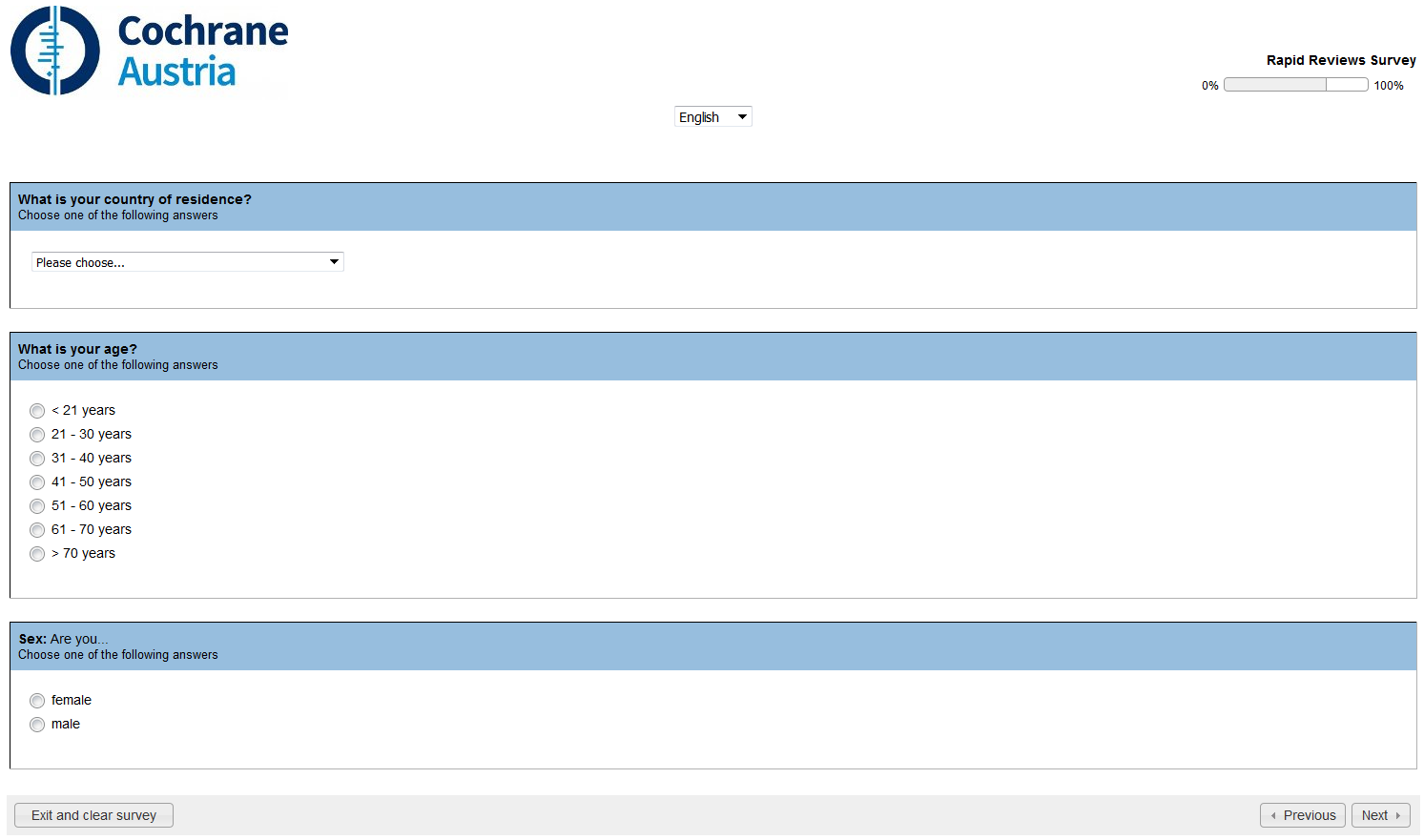


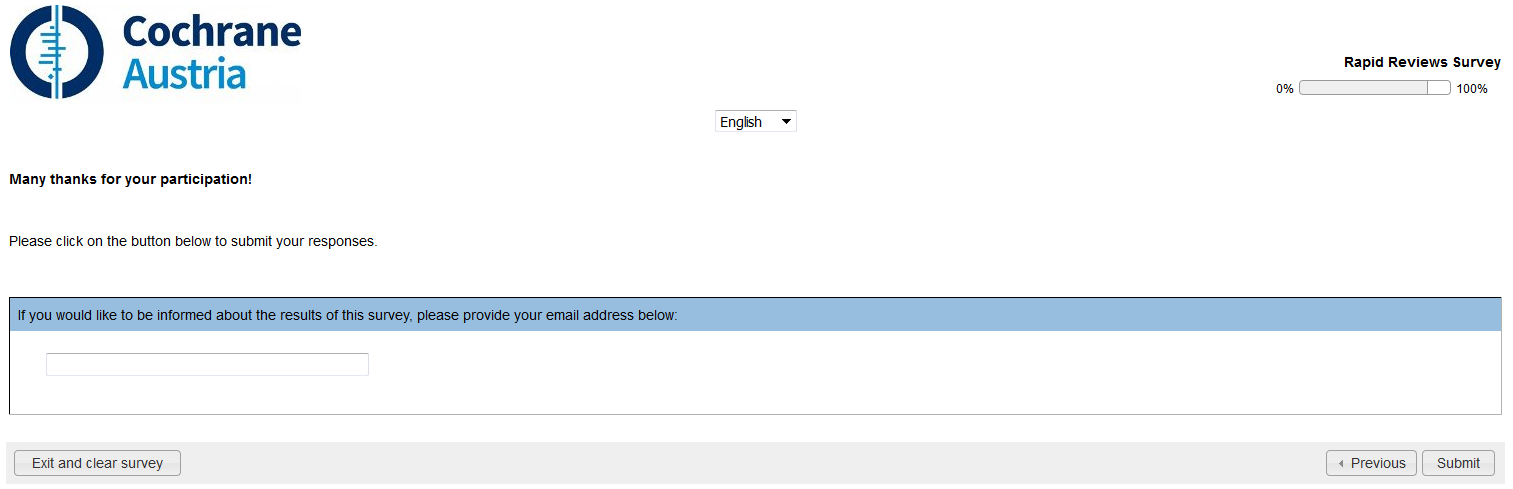

Supplement: Supplementary file 3 — Questionnaire in English. This file contains screenshots of the questionnaire in English. (DOCX 709 kb) [file 12874_2017_406_MOESM3_ESM.docx]
