# Supplementary material for: Trading certainty for speed - how much uncertainty are decisionmakers and guideline developers willing to accept when using rapid reviews: an international survey
Source: BMC Med Res Methodol. 2017 Aug 14;17:121. doi: 10.1186/s12874-017-0406-5 (PMC5557322; doi:10.1186/s12874-017-0406-5)
Supplement: Supplementary file 2 — Recruitment e-mail. This file contains the text of the recruitment e-mail in English for individually contacted decisionmaker and guideline developer. (DOCX 16 kb) [file 12874_2017_406_MOESM2_ESM.docx]

**Additional file 2**

**Recruitment e-mail in English for individually contacted decisionmaker and guideline developer**

Dear Dr. [*name of participant*]

We are contacting you as a guideline developer [replace with “decisionmaker” if necessary] in an effort to help improve Rapid Reviews.

Although **Systematic Reviews** offer the most reliable and valid support for decisionmaking they can take a long time to complete and users often need information fast. For this reason **Rapid Reviews**, which produce results quicker, have become a pragmatic alternative. A potential trade-off, however, is the lower level of reliability of results for Rapid Reviews. Wrong answers might lead to an increased risk of making incorrect decisions or recommendations.

**How much risk of getting a wrong answer are you willing to accept in exchange for a rapid synthesis of evidence?** This is the question our survey hopes to answer.

Would you please take the linked **10-minute survey to assist Cochrane in this Rapid Review study**?

<http://survey.cochrane.at/index.php/267551/lang-en>

Our aim is to reach a wide range of experts and decisionmakers. **Please consider forwarding this email to friends and colleagues that use evidence synthesis for decision making** for their participation**.**

If you have any questions or feedback regarding this survey, please contact [judith.greimel@cochrane.at](mailto:judith.greimel@cochrane.at).

Best wishes,

Gerald Gartlehner, MD, MPH

Director of Cochrane Austria

Barbara Nussbaumer, MSc BSc

Associate Director of Cochrane Austria
